# ASO Author Reflections: Sentinel Node Navigated Surgery as a New Treatment Strategy for High-Risk T1 Esophageal Adenocarcinoma

**DOI:** 10.1245/s10434-023-13382-x

**Published:** 2023-04-04

**Authors:** Charlotte N. Frederiks, Bas L. A. M. Weusten

**Affiliations:** 1 Department of Gastroenterology and Hepatology, St. Antonius Hospital, Nieuwegein, The Netherlands; 2 Department of Gastroenterology and Hepatology, University Medical Center Utrecht, Utrecht University, Utrecht, The Netherlands

## Past

While endoscopic resection is the first-choice treatment for early esophageal adenocarcinoma (EAC), adjuvant surgery might be indicated depending on the presence of high-risk features associated with lymph node metastases (LNM). These high-risk features include deep submucosal invasion, poor tumor differentiation, and presence of lymphovascular invasion. On the basis of current guidelines, most patients with a high-risk T1 submucosal (T1b) cancer are treated with additional esophagectomy since LNM rates of up to 37% have been reported.^1^ For high-risk mucosal (T1a) cancer, no clear consensus exists on the best treatment option, and some patients are referred for surgery, since the incidence of LNM varies up to 20%.^2^ However, these relatively low risks of LNM might dispute the need for immediate adjuvant surgery in patients with high-risk T1 EAC, especially considering esophagectomy is a major surgical procedure with significant morbidity and reduced quality of life postoperatively.^3^

## Present

A less invasive alternative for these specific patient categories might be a selective lymphadenectomy using sentinel node navigated surgery (SNNS), after which additional treatment can be tailored on the basis of sentinel lymph node involvement.^4^ This prospective, multicenter pilot study is the first to investigate the feasibility and safety of a new treatment strategy in ten patients consisting of radical endoscopic resection of a high-risk T1 EAC followed by tailored lymphadenectomy using SNNS. Localization and dissection of sentinel nodes was feasible in all patients (median of three sentinel nodes per patient) without any major complications, while two patients (20%) were found to have a metastasis in one of the resected sentinel nodes.^5^ Therefore, this minimally invasive treatment strategy appears to be a feasible and safe instrument to tailor lymphadenectomy, preserve the esophagus, and thus personalize additional treatment on the basis of lymph node involvement in carefully selected patients

## Future

Despite these promising short-term results, the exact position of this new esophageal preserving strategy in the treatment algorithm for high-risk T1 esophageal cancer needs to be studied in future research with long-term follow-up. Ideally, this new treatment strategy with SNNS will be offered to a selected subgroup of patients with high-risk T1 EAC who are most likely to benefit from SNNS. One of the challenges, however, is to reliably identify these “high-high risk” patients. In addition, the superiority of SNNS over a watchful waiting strategy, in which patients after endoscopic resection of a high-risk T1 EAC are followed intensively with 3-monthly upper endoscopy plus endoscopic ultrasound, with additional surgery only in case of proven LNM, remains to be shown.
